# Impact of asthma control on quality of life among palestinian children

**DOI:** 10.1038/s41598-025-91756-9

**Published:** 2025-02-27

**Authors:** Ali Aldirawi, Ahmad R. Al-Qudimat, Tamara Al Rawwad, Fadwa Alhalaiqa, Abdallah Alwawi, Yan Jin, Samer Abuzerr, Eman Hammad, Lina Rjoub

**Affiliations:** 1 Xiangya School of Nursing, Central South University, Changsha, Hunan Province China; 2 Department of Public Health, College of Health Sciences, Qatar University, Doha, Qatar; 3 Department of Social Work, School of Applied Humanities and Social Sciences, German Jordanian University, Amman, Jordan; 4 Pre-Clinical Affairs, College of Nursing, Qatar University, Doha, Qatar; 5 Nursing Department, Faculty of Health Professions, Al-Quds University, Abu Dies, Palestine; 6 Nursing Department, Third Xiangya Hospital, Central South University, Changsha, China; 7 Department of Medical Sciences, University College of Science and Technology, Khan Younis, Gaza, Palestine; 8 Hebron Governmental Hospital, Al-Quds University, Abu Dies, Palestine; 9 Hebron Governmental Hospital, Hebron, Palestine

**Keywords:** Asthma control, Quality of life, Children, Asthma, Palestine, Diseases, Health care, Medical research, Risk factors, Signs and symptoms

## Abstract

Asthma is a chronic respiratory disease that significantly affects children, impacting their health-related quality of life. This study aimed to explore the relationship between asthma control and quality of life among pediatric asthma patients in the West Bank, Palestine. A descriptive, cross-sectional study was conducted among 220 pediatric patients with asthma and their mothers, recruited from four governmental hospitals. Data were collected using self-administered questionnaires that included demographic information, an asthma control test, and the Pediatric Asthma Quality of Life questionnaire. The final analysis included 182 children with a mean age of 8 years. The results showed that 71.9% of the children had uncontrolled asthma, with poor health-related quality of life reported by approximately 70% of this group. In contrast, only 2.7% of children with controlled asthma reported reduced quality of life. The findings indicate a strong association between asthma control and health-related quality of life. Healthcare policies should prioritize educational programs for children and their parents to improve asthma management and overall well-being.

## Introduction

Asthma is a prevalent chronic inflammatory condition that significantly impacts the lives of pediatric patients, their families, and healthcare systems worldwide^1^. Affecting approximately 14% of children globally, asthma’s incidence and severity have escalated over the past three decades, as evidenced by rising hospitalization rates and asthma-related mortality^2^. This respiratory condition poses particular challenges for children, where its effects on physical, social, and emotional well-being contribute to reduced quality of life (QoL) and increased healthcare needs^3^. Asthma prevalence among children in the Middle East varies considerably, with reports showing rates from 0.7% in Iran to 22.3% in Iraq. In Palestine, pediatric asthma prevalence differs by location: 17.1% in villages, 8.8% in cities, and 9.4% in refugee camps, reflecting the influence of socio-environmental factors^4^.

Health-related quality of life (HRQoL), which considers the impact of health on functioning and well-being, is particularly relevant for children with asthma, as uncontrolled symptoms can hinder their physical, social, and emotional development. Asthma significantly contributes to morbidity, reduced HRQoL, and increased healthcare costs. However, asthma detection and management remain suboptimal, particularly in low-income countries^5,6^. Guidelines emphasize adjusting treatment primarily based on asthma control, underscoring the need to understand the link between asthma management and HRQoL among pediatric patients in challenging settings^7^.

Asthma impacts various aspects of children’s lives, potentially leading to depression, academic underachievement, behavioral issues, cognitive disorders, insomnia, and difficulties with internalizing and externalizing behaviors^8^. Social limitations, such as avoiding sleepovers and friends with pets, further restrict the lives of children with asthma^9–11^. Evaluating the impact of asthma control on HRQoL is vital for understanding disease burden, identifying risk factors, and informing public health strategies. Such assessments support strategic planning, informed resource allocation, and the evaluation of the effectiveness of healthcare interventions^12^. Children with well-controlled asthma experience fewer symptoms and reduced medication reliance^13^. Research has shown that uncontrolled asthma, asthma severity, and prior hospitalization are associated with poor HRQoL among children and adolescents^14^. Children with both managed and uncontrolled asthma experienced similar psychosocial impacts, with no correlation observed between asthma control and psychosocial well-being^15^.

Understanding the unique challenges and factors influencing asthma control in Palestinian territories is essential because the sociopolitical and environmental conditions impact health outcomes and access to care. Resource scarcity, fragmented systems, and barriers to medication availability characterize the healthcare environment in these areas. Additionally, risk factors such as pollution, exposure to chemical and biological agents, and stress from conflict and displacement necessitate targeted interventions and evidence-based solutions. Asthma management in Palestine is complicated by socio-political and environmental factors, such as limited healthcare resources, pollution, and high stress from conflict, affecting asthma control and HRQoL. Although asthma greatly impacts children in Palestine, limited research explores asthma control’s effect on HRQoL using standardized guidelines. This study aims to address this gap by assessing the impact of asthma control on HRQoL among pediatric patients in the West Bank, aligning with GINA guidelines for consistency and reliability^16,17^. This study aimed to assess the impact of asthma control on the overall HRQoL among pediatric patients with asthma in the West Bank of Palestine.

## Methods

### Study design and setting

This descriptive, cross-sectional study was conducted between January and March 2024 on children diagnosed with asthma at governmental hospitals in the West Bank in Palestine. The participants were recruited from four governmental hospitals in the West Bank, namely, Governmental Hebron Hospital, Governmental Beit Jala Hospital in Hebron governorate, Governmental Yatta Hospital, and Governmental Durra Hospital from Bethlehem governorate. These hospitals provide comprehensive pediatric care and serve large populations of children with asthma. To ensure a representative regional sample, Hebron and Bethlehem governorates were randomly selected from 11 West Bank governorates in Palestine.

### Participants

Children aged 5 to 14 years diagnosed with asthma at least six months prior to the study, as per Global Initiative for Asthma (GINA) guidelines, were eligible to participate. Exclusion criteria included pre-existing medical conditions that could influence results, age under 5 or over 14, or an asthma diagnosis within six months of the study^18^. A six-month duration was chosen to provide sufficient time to ascertain the control level of the children’s asthma^19^. Eligible children were randomly selected from hospital records, and their primary caregivers, typically mothers, were approached for participation. After being informed of the study’s objectives, all participants provided signed consent, including assent from children where appropriate.

### Sample size

According to the Palestinian Ministry of Health^20^, approximately 500 pediatric asthma patients require visits to governmental hospitals for monitoring. The sample size was determined via Cochran’s formula with the online website link (http://www.raosoft.com*)* with the following criteria: a significance level of 0.05, a confidence interval of 95%, and a response distribution of 50%. A simple random selection technique was employed to enlist 220 mothers with pediatric asthma via a computerized system in these hospitals.

### Instruments used in the study

Data were collected through a self-administered survey of mothers of pediatric patients with asthma. The questionnaire included three parts: sociodemographic and clinical data, the Asthma Control Test (ACT), and the Pediatric Asthma Quality of Life Questionnaire (PAQLQ)^13,21,22^. The sociodemographic and clinical data included the child’s age, sex, family history of asthma, prior mothers’ participation in health education programs about asthma, and use of asthma control medications including Inhaled corticosteroids (ICS), long-acting beta2-agonists (LABA), leukotriene receptor antagonists (LTRA), and tiotropium bromide. The ACT is a validated and globally recognized asthma control test^13,21^. The scale includes a 5-point Likert scale to evaluate the signs of asthma during either the daytime or nighttime and the impact of asthma disease on everyday actions over the past four weeks, with scores ranging from 5 (indicating inadequate management of asthma) to 25 (indicating optimal management of asthma)^13^. The ACT assesses daytime and nighttime asthma symptoms, the use of relief medication, and limitations in daily activities in the previous four weeks. ACT scores > 19 indicate controlled asthma, and those < 19 indicate uncontrolled asthma^23^. This tool [English] has demonstrated strong convergent validity for the ACT, with correlation coefficients ranging from 0.71 to 0.92^24^. The ACT has been translated into Arabic and validated via the Tunisian [Arabic] language, with an acceptable Cronbach’s alpha of 0.85 ^25^.

The PAQLQ is a standardized measure of children’s HRQoL. It has 23 items that cover three fields: emotional function, symptoms, and activity limitation^24^. It consists of 5 questions that measure distress actions, 10 that examine the discomfort resulting from asthma episodes, and 8 that ask about the emotional impact of asthma by evaluating feelings of frustration, fear, annoyance, or upset experienced by patients^24,26^. The responses are evaluated via a 7-point Likert scale ranging from 1 (indicating the most significant impairment) to 7 (representing no impairment). The overall score, which reflects the HRQoL, is calculated by finding the mean of the answers to the 23 questions. A higher score implies a better HRQoL. The overall score varied from 23 to 161. The English version of the PAQLQ has demonstrated strong validity, with correlation coefficients ranging from 0.73 to 0.92^27^ and a Cronbach’s alpha value of 0.83 ^26^. The [Arabic] version of the PAQLQ was validated (Cronbach’s alpha 0.84)^28^.

### Data collection

Data were collected from mothers of pediatric patients with asthma through two research assistants, who are medical doctors (MDs) and have many years of experience in children’s medical care and academic data collection techniques. An online training session was organized to explain the goals of the investigation and provide guidance on data collection following hospital regulations and standardized scientific procedures. The mothers were fully informed of the purpose of the investigation before being invited to participate in the study. Those who agreed to participate provided their written consent before completing the questionnaire, which took approximately 20 min.

### Ethical consideration

This investigation acknowledges ethical approval from the Institutional Review Board (IRB) of Central South University in China under reference number [E202404]. The Ministry of Health in Palestine also obtained formal permission to collect data. A written consent form was systematically issued to every mother of a pediatric patient with asthma. In adherence to ethical standards, explicit personal agreement was sought from each child participant, accompanied by a clear statement affirming their right to decline participation, abstaining from answering any questions, and withdrawing from the study at any point.

### Data analysis

The statistical analysis was performed via SPSS software (version 25). Descriptive statistics, including means and standard deviations for continuous variables and percentages for categorical variables, were generated. The bivariate associations between categorical variables were assessed via the chi-square test, whereas differences in continuous variables were examined via the independent t test and analysis of variance (ANOVA). Multivariate logistic regression analysis was conducted to investigate the associations between sociodemographic variables and HRQoL. Variables that demonstrated a significance level of *p* < 0.25 in the univariate analysis were selected for inclusion in the multivariable regression analysis. Univariate logistic regression analysis revealed an association between asthma control and HRQoL. This analysis provided 95% confidence intervals (CIs) and *p value* estimates. The statistical significance of the associations was established as *p* < 0.05.

## Results

### Demographic characteristics of the participants

The study sample consisted of children aged 5–14 years who had been diagnosed with asthma by a specialist at least six months before the commencement of the study. Among the 220 patients approached, 182 consented to participate, resulting in a response rate of 83%. The mean age of the participants was 8 (± 2.2) years, with 104 (57.1%) males. A significant proportion of the mothers (85, 46.7%) had attained a university education. The mean duration since asthma onset among all participants was 17.6 (± 3.1) months. Clinically, 153 (84.1%) of the participants had not engaged in health education programs related to asthma.

A significant association was found between mothers’ education levels and asthma control (*p* = 0.04), with higher mothers’ education associated with better-controlled asthma. Additionally, pediatric patients with uncontrolled asthma were significantly more likely to have had at least two hospitalizations in the previous month due to asthma than were children in the controlled asthma group were (*p* < 0.001). Notably, children in the controlled asthma group were more likely not to use medication (*p* < 0.001) than those in the uncontrolled asthma group were. Furthermore, pediatric patients with uncontrolled asthma were associated with school absenteeism (*p* < 0.001) (Table 1).

**Table 1:** Sample characteristics and associations with pediatric asthma status.

Variables	Uncontrolled asthma (137)	Controlled asthma (45)	All patients (182)	*p* value
**Age (years)**	8.0 (± 2.2)	9.0 (± 2.4)	8.0 (± 2.2)	0.27
**Gender** Male Female	82(59.9%) 55(40.1%)	22(48.9%) 23(51.1%)	104 (57.1%) 78 (42.9%)	0.20
**Duration of Asthma Onset (months)**	17.0(± 3.1)	19.0(± 3.3)	17.6(± 3.1)	0.96
**Family history of asthma** No Yes	100(73.0%) 37(27.0%)	28(62.2%) 17(38.8%)	128 (70.3%) 54 (29.7%)	0.17
**Mothers’ education** Primary Secondary University	21(15.3%) 56(40.9%) 60(43.8%)	1(2.2%) 19(42.2%) 25(55.6%)	22 (12.1%) 75 (41.2%) 85 (46.7%)	0.04*
**Severity of Asthma** Mild Moderate Severe	9(6.6%) 62(45.3%) 66(48.2%)	32(71.1%) 13(28.9%) 0(0.0%)	41 (22.5%) 75 (41.2%) 66 (36.3%)	< 0.001*
**Emergency department visit last month** No Once Twice Three times > 3 time	27(19.7%) 8(5.8%) 32(23.4%) 44(33.1%) 26(19.8%)	28(62.2%) 13(28.9%) 4(8.9%) 0(0.0%) 0(0.0%)	55(30.2%) 21(11.5%) 36(19.8%) 44(24.2%) 26(14.3%)	< 0.001*
**Use control medications** No Yes	91(66.4%) 46(33.6%)	40(88.9%) 5(11.1%)	131 (72.0%) 51 (28.0%)	0.004*
**School absenteeism last month** No Yes	46(33.6%) 91(66.4%)	41(91.1%) 4(8.9%)	87 (47.8%) 95 (52.2%)	< 0.001*
**Participation in Health Teaching Programs** No Yes	112(81.8%) 25(18.2%)	41(91.1%) 4(8.9%)	153 (84.1%) 29 (15.9%)	0.14

* Data are significant with a *p* value < 0.05.

### Level of asthma control

**Fig. 1:**
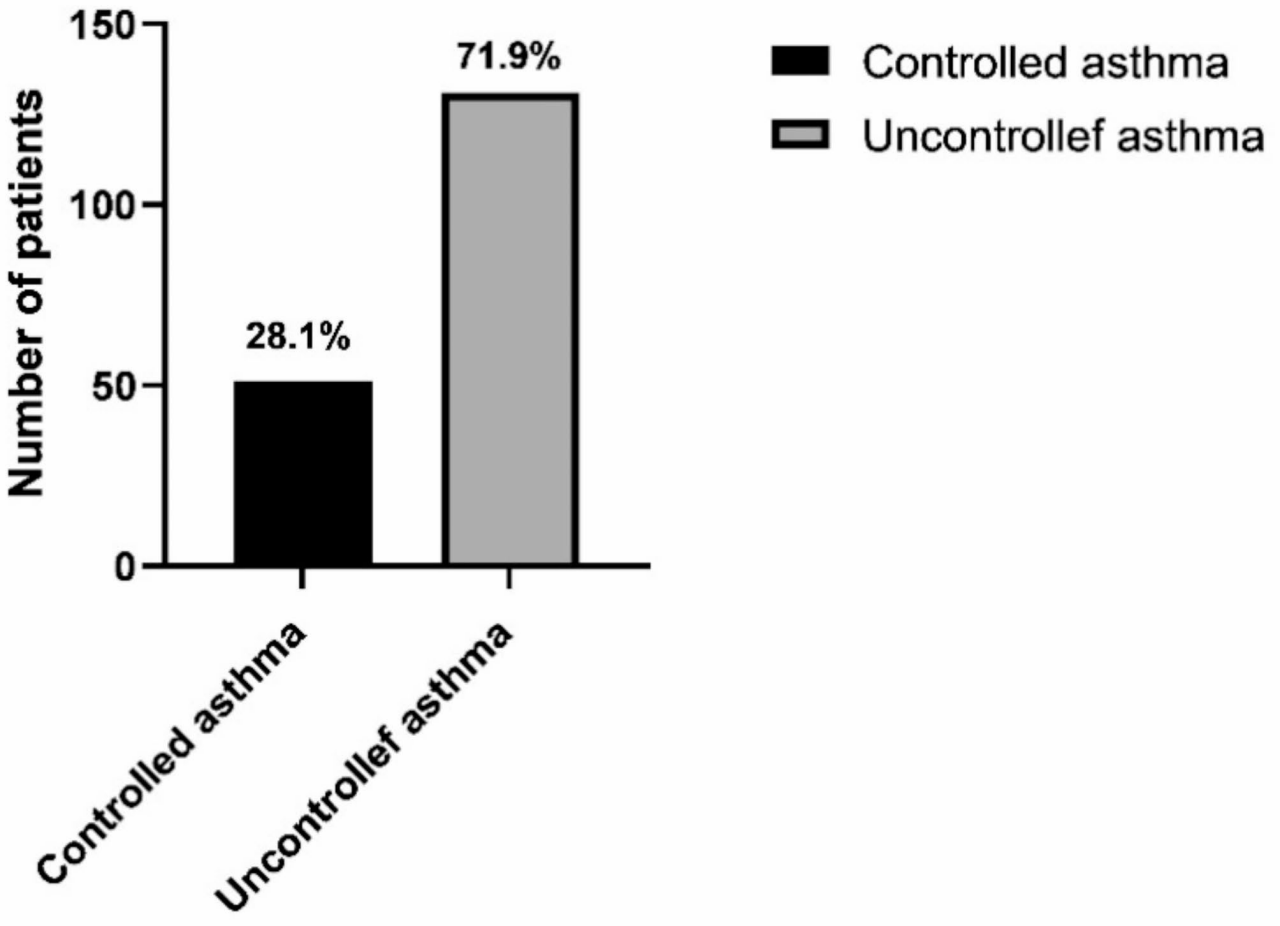
Asthma control levels.

The participants were divided into two groups on the basis of their ACT scores: controlled asthma (ACT score > 19) and uncontrolled asthma (ACT score < 19). The mean ACT score among children was 14.13 (± 5.23), indicating that the majority had uncontrolled asthma. Specifically, 71.9% (131 out of 182) of the participants had uncontrolled asthma, whereas 28.1% (51 out of 182) had controlled asthma **(**Fig. 1**)**.

### Pediatric asthma quality of life questionnaire (PAQLQ) scores

The analysis of the PAQLQ scores revealed that the highest score of 4.37 (± 1.52) was reported for the item related to physical activities, such as running, swimming, sports, walking uphill or upstairs, and bicycling. Conversely, the lowest score of 3.64 (± 1.62) was reported for the item “feeling out of breath due to your child’s asthma.” The patients’ HRQoL scores ranged from 1 to 7, with a mean average score of 3.91 (± 1.61), highlighting the poor HRQoL experienced by pediatric patients with asthma (Table 2).

**Table 2:** Distribution of patient PAQLQ scores (*N* = 182).

Questions	Mean	SD
**How bothered has your child been during the last week doing:**		
Physical activities (running, swimming, sports, walking uphill/upstairs and bicycling)?	4.37	1.52
Are we with animals (such as playing with pets and looking after animals)?	4.14	1.72
Activities with friends and family (such as playing at recess and doing things with your child’s friends and family)?	3.98	1.67
Coughing	3.97	1.55
Asthma attacks	3.98	1.58
Wheezing	3.98	1.64
Tightness in the chest of your child	3.92	1.61
Shortness of breath	3.94	1.59
How much were you bothered by your child’s asthma doing these activities?	3.99	1.57
**In General, how often during the last week did your child:**		
My child feels frustrated because of her/his asthma.	3.95	1.63
My child feels tired because of her/his asthma.	3.86	1.61
My child feels worried, concerned, or troubled because of her/his asthma.	3.83	1.60
Does your child feel angry because of her/his asthma?	3.84	1.49
My child feels irritable (cranky/grouchy) because of her/his asthma.	3.90	1.69
Does your child feel different or left out because of her/his asthma?	3.82	1.58
My child feels frustrated because he/she cannot keep up with others.	3.92	1.56
My child wakes up during the night because of her/his asthma.	3.81	1.66
My child feels uncomfortable because of her/his asthma.	3.83	1.57
My child feels out of breath because of her/his asthma.	3.64	1.62
My child cannot keep up with others because of her/his asthma.	3.74	1.60
My child had trouble sleeping at night because of her/his asthma.	3.97	1.54
My child feels frightened by an asthma attack.	3.87	1.79
My child had difficulty taking a deep breath.	3.86	1.64
**Average of HRQoL score**	**3.91**	**1.61**

*SD*: Standard Deviation, HRQoL: Health-related Quality of Life.

### Correlation between control medications use and study outcomes

Further analysis examined the correlation between asthma control medications and asthma control level, and HRQoL scores. Children using control medications including inhaled corticosteroids (ICS), long-acting beta2-agonists (LABA), leukotriene receptor antagonists (LTRA) and tiotropium bromide demonstrated moderately significant correlations with better asthma control (*r* = 0.615, *p* < 0.001) and improved HRQoL scores (*r* = 0.676, *p* < 0.001) (Table 3).

**Table 3:** Correlation analysis between control medications use and asthma control and HRQoL

**Variable**		**Asthma Control**		**HRQoL**	
Use asthma control medications including ICS, LABA, LTRA, and tiotropium bromide	Pearson Correlation	0.615^*^		0.676^*^	
	*p*-value	**< 0.001**		**< 0.001**	
	N	182		182	

* Correlation is significant at the *p* < 0.01 level.

### Associations between the outcome variables

A notable disparity was observed in the HRQoL among pediatric patients on the basis of their asthma control status. Approximately 70.3% of patients with uncontrolled asthma reported poor HRQoL, in stark contrast to only 2.7% of those with controlled asthma who experienced diminished HRQoL. The likelihood of having uncontrolled asthma was nearly fourteen times greater for individuals with poor HRQoL than for those with good HRQoL (Table 4).

**Table 4:** Univariate logistic regression analysis of asthma status with HRQoL.

Health-related Quality of Life	Asthma Status		COR (95%CI)	*p*-value
	Uncontrolled *N* (%)	Controlled *N* (%)		
**Good Health-related Quality of Life**	9 (5%)	40 (22%)	0.008(0.002–0.027)	< 0.001*
**Poor Health-related Quality of Life**	128 (70.3%)	5 (2.7%)	1.00	
**Total**	137	45	4.37(2.02–9.45)	< 0.001*

* Data are significant with *p* value < 0.05, QoL; Quality of Life, COR: Crude odds ratio, CI: Confidence interval.

A logistic regression analysis explored the associations between sociodemographic variables and HRQoL. Before analysis, the predictor variable, HRQoL, underwent a preassessment to confirm compliance with the assumption of linearity in logistic regression. The predictor variable was found to contribute significantly to the model. The logistic regression analysis revealed that sex, family history of asthma, family size, presence of other chronic diseases, duration since asthma onset, and participation in asthma-related health education programs were not significantly associated with poor HRQoL. In contrast, age, parental marital status, asthma severity, and medication use were significantly associated. Compared with those with married parents, children with divorced parents presented markedly reduced odds of having good HRQoL, with a 94% decrease in the odds of having poor HRQoL (AOR = 0.06; 95% CI: 0.01–0.43; *p*-value = 0.005). Patients with moderate asthma severity had significantly lower odds of having good asthma-related HRQoL than those with mild or severe asthma did, with a 98% decrease in the odds of having poor HRQoL (AOR = 0.02; 95% CI: 0.004–0.11; *p-value* = 0.001). Individuals who did not use medication had a reduced likelihood of experiencing diminished HRQoL (COR = 0.21; 95% CI: 0.07–0.58; *p-value* = 0.002) (Table 5).

**Table 5:** Multivariable logistic regression analysis of factors associated with HRQoL.

	Health-related Quality of Life		COR (95%CI)	AOR (95%CI)	*p* value
	Good HRQoL *N* (%)	Poor HRQoL *N* (%)			
**Age category** 4–6 7–10 11–14	9 19 21	34 36 36	1 1.13(0.46–2.87) 2.20(0.88–5.49)	1 3.8(0.62–24.14) 3.3(0.63–17.32)	0.77 0.09
**Gender** Male Female	26 23	78 55	1 1.25(0.64–2.42)	1 1.27(0.41–3.95)	0.50
**Parents marital status** Married Divorced Widowed	44 1 4	116 12 5	1 0.21(0.02–1.74) 2.1(0.53–8.24)	1 0.06(0.01–0.43)* 3.02(0.42–21.57)	0.005* 0.27
**Family members** Less than 4 4–7 More than 7	11 29 9	45 64 24	1 1.85(0.83–4.1) 1.53(0.55–4.22)	1 0.79(0.22–2.8) 1.7(0.41–6.99)	0.72 0.45
**Family history of asthma** No Yes	23 17	96 37	1 1.37(0.68–2.78)	1 1.45(0.46–4.56)	0.52
**Other chronic disease** No Yes	45 4	114 19	1 0.53(0.17–1.65)	1 0.41(0.07–2.14)	0.21
**Duration of asthma (m)** 1–10 11–20 21–30 More than 30	21 15 4 9	72 24 15 22	1 2.14(0.95–4.81) 0.91(0.27–3.06) 1.40(0.56–3.51)	1 3.06(0.61–5.4) 1.83(0.26–12.88) 1.90(0.31–11.62)	0.06 0.54 0.48
**Severity of asthma** Mild Moderate Severe	34 15 0	7 60 66	1 0.05(0.01–0.13)* 1	1 0.02(0.004–0.11)* 1	0.001*
**Using control medications** No Yes	44 5	87 46	1 0.21(0.07–0.58)*	1 0.47(0.09–2.43)	0.002*
**Participate in health teaching programs about asthma**. No Yes	44 5	109 24	1 0.51(0.18–1.44)	1 0.78(0.22–2.76)	0.700

AOR = adjusted odds ratio, COR = crude odds ratio; *Statistically significant at *p* < 0:05.

## Discussion

This study revealed that controlling asthma has a positive effect on children’s HRQoL, with a significant percentage of children having uncontrolled asthma. Pediatric patients with asthma, particularly those with uncontrolled asthma, experience significantly diminished HRQoL. Our study revealed that most participants had uncontrolled asthma, which aligns with findings of previous research studies indicated the pediatric asthma patients had low levels of asthma control^14,29^. The well-being of Palestinian pediatric patients with asthma appears to be affected by various factors, including socioeconomic challenges such as limited healthcare access and economic hardships, compounded by environmental factors such as air pollution and allergies^30–32^. Furthermore, these findings were consistent well with other studies that showed there was association between asthma control and HRQoL among pediatric asthma patients^14,33,34^. The children with uncontrolled asthma reported significantly lower HRQoL and there is an association between asthma control and physical and emotional well-being^35,36^. Another research concluded that asthmatic children and adolescents who effectively controlled their asthma exhibited better HRQoL^37^.

Uncontrolled pediatric asthma was associated with at least two hospitalizations in the past month in current study. Furthermore, we noted a significant correlation between uncontrolled Palestinian pediatric asthma and school absenteeism for many reasons. Socioeconomic difficulties and deficiencies in health education may impede early intervention and ongoing follow-up treatment. In educational institutions, environmental factors including dust and inadequate air quality can exacerbate asthma symptoms, leading to increased school absenteeism. The combined effect of these variables not only hinders the child’s education but also imposes a considerable strain on families and healthcare systems, highlighting the necessity for enhanced asthma management programs and community education campaigns in Palestine. This finding is consistent with another study reported that children hospitalized for asthma had lower scores in HRQoL. The poor asthma control leads to frequent symptoms, decreased physical activity, and negatively impacts emotional well-being^38^. Similarly, a study in Australia reported a significant correlation between the frequency of hospital admissions and a low HRQoL in pediatric patients with asthma^39^. The hospital admissions and school absenteeism are closely associated with poor HRQoL in pediatric patients with asthma^17,38–40^. Concerning asthma management and education, our study did not find a significant correlation between parental participation in asthma-related health education programs and asthma control levels. However, international guidelines widely recommend these programs to improve asthma management. This finding is consistent with the research study that has shown that disparities in healthcare professionals’ provision of asthma action plans might contribute to suboptimal asthma control outcomes^41^.

Our findings indicated that pediatric patients with uncontrolled asthma had higher rates of emergency department visits, which is consistent with the findings of previous studies that linked poor asthma control to exacerbations and increased healthcare utilization^42,43^. The importance of psychological functioning and feeling depressed was noted, indicating that Palestinian pediatrics with uncontrolled asthma may experience more difficulties in these aspects of HRQoL than those with controlled asthma independently. Another research has shown that pediatric with asthma often have more other health conditions and report greater levels of psychological distress^44^. Several epidemiological studies have indicated that asthma inadequately treated is linked to a decrease in overall HRQoL, as measured by generic indicators^45–48^. The impact of asthma control levels on HRQoL was examined in two studies. Both studies revealed that moderately managed and uncontrolled asthma was significantly associated with worse HRQoL in both symptom-based asthma^45^ and self-reported clinician diagnoses of asthma^49^.

Our results found the correlation between asthma control medications and asthma control level, and HRQoL scores. The Palestinian children using control medications including ICS, LABA, LTRA, and tiotropium bromide demonstrated moderately significant correlations with better asthma control and improved HRQoL scores. The control medications are crucial for the sustained control of asthma, mitigating airway inflammation and averting exacerbations. Considering that inadequately controlled asthma is a primary contributor to emergency visits and school absences, adherence to these drugs markedly improves symptom management and HRQoL. Although patients adhere to control medications such as ICS/LABA, they have higher symptom burden and worse HRQoL^50^.

Biologic therapies, such as omalizumab and dupilumab, have shown significant benefits in reducing exacerbation rates, improving asthma control, and enhancing the quality of life (HRQoL) in children with uncontrolled asthma^51^. These medications work by targeting specific inflammatory pathways, offering a personalized treatment approach for patients with eosinophilic or allergic asthma^50^. However, despite their proven efficacy, biologics remain largely inaccessible in Palestine, mainly due to high costs, limited availability, and healthcare infrastructure challenges. In many low-resource settings, including Palestine, asthma management relies heavily on ICS and LTRA with biologics being reserved only for severe, treatment-resistant cases^5^. Given these challenges, future research and policy discussions should explore the feasibility of introducing biologic therapies into clinical practice in Palestine, balancing cost-effectiveness with patient needs^14^. While our study did not include biologic agents due to these limitations, we recognize their potential future role in improving asthma outcomes in Palestinian children. Addressing economic and logistical barriers to biologic access could be an important step toward optimizing asthma care in the region.

Our finding found the pediatric asthma who did not use control medication, diminished quality of life. In Palestine, the restricted access to healthcare resources, especially asthma management drugs, may significantly impact places with economic limitations or inadequate healthcare infrastructure. A lack of understanding or education among carers on the significance of consistent administration of control drugs may hinder effective asthma treatment. Cultural and socioeconomic issues, such dependence on emergency treatment instead of preventive strategies, may further aggravate the problem. Moreover, the psychological and physical strain of unmanaged asthma symptoms, such as recurrent exacerbations, absenteeism from school, and diminished engagement in physical and social activities, directly affects the child’s overall well-being and quality of life. These findings were consistent with other studies that found the compliance of pediatric asthma in the proper use of control medications, possession of inhalers during a child’s acute respiratory distress were associated of a clinically significant improvement in quality of life^52–55^.

The limitations of this study are noteworthy and should be considered when the findings are interpreted. The data collection relied on self-administered questionnaires, introducing the possibility of recall bias. Participants may have inaccurately recalled or misrepresented their asthma control status or HRQoL, potentially influencing the reliability of the results. Additionally, social desirability bias might have affected responses, as participants could overstate their adherence to treatment regimens. This study cannot establish causal relationships between the variables investigated. While associations between demographic factors, asthma control, and HRQoL have been identified, longitudinal studies are needed to confirm these relationships over time and explore causality. Finally, one of the limitations of this study is the inability to evaluate the impact of biologic treatments on asthma control and HRQoL in this population. Future research should explore the feasibility of integrating biologic therapies into asthma management in Palestine, considering the economic and logistical challenges that may hinder their widespread implementation.

## Conclusion

This study revealed that a significant portion of pediatric patients experienced uncontrolled asthma and poor HRQoL. The association between poor HRQoL and uncontrolled asthma, along with frequent emergency department visits in the past month, highlights the critical need for targeted interventions to enhance asthma management and overall well-being in this vulnerable Palestinian population. This approach should encompass medical management and considerations for psychological impacts, social, emotional and potential behavioral issues. Healthcare professionals must priorities activities that improve access to asthma control drugs and reinforce asthma education programs for pediatric patients with asthma and their caregivers. Executing community-based interventions, including school health initiatives and regular consultations with specialized asthma care teams, can diminish hospitalizations and school absences. Furthermore, tackling environmental causes via public health initiatives and enhanced living circumstances might significantly mitigate the incidence of asthma. These initiatives seek to advance asthma management, improvement overall HRQoL for children with asthma, and decrease the long-term healthcare expenses associated with mismanaged chronic illnesses. Future research should investigate the impediments to successful asthma management which encompassing socioeconomic, environmental, and healthcare system factors. Longitudinal studies are essential to assess the enduring effects of educational and intervention programs on asthma management and HRQoL. Furthermore, research ought to examine the efficacy of community-based efforts, including school-based asthma management programs in enhancing outcomes for pediatric asthma patients. Subsequent research may investigate the impact of environmental alterations and policy reforms on mitigating asthma triggers and hospital admissions. These initiatives will provide significant information for formulating focused strategies to improve asthma treatment and support in resource-constrained environments.

## Data Availability

The data analyzed during this study are available from the corresponding author upon reasonable request.
